# Situational analysis of service provision for adolescents with mental and neurological disorders in in two districts of Ghana

**DOI:** 10.1186/s13033-021-00457-z

**Published:** 2021-04-15

**Authors:** Adrienne Formentos, Kenneth Ayuurebobi Ae-Ngibise, Solomon Nyame, Kwaku Poku Asante

**Affiliations:** 1 National Academies of Sciences, Engineering, and Medicine, Washington, DC 20001 USA; 2 Kintampo Health Research Centre, Ghana Health Service, P.O. Box 200, Kintampo, Ghana

**Keywords:** Situational Analysis, Mental health services, Adolescents, Rural Ghana

## Abstract

**Background:**

Prevalence among adolescents with mental disorders are about 20% worldwide. In 2012, Ghana enacted the Mental Health Act, Act 846 to regulate mental health care, but did not include specific programmatic details of service provision nor any measurable indicators for adolescent mental health. Currently no service programmes focused on adolescents and no aggregated data exists documenting prevalence of mental and neurological disorders among adolescents. In the Brong Ahafo region, mental health providers carry out simultaneous programmes to diagnose, treat, and counsel patients. There is a need to investigate how these service programmes are currently functioning as measured by World Health Organisation guidelines. This study therefore, investigated quality of service provision for adolescents with mental disorders in Kintampo North and South districts of central Ghana.

**Methods:**

Mixed method approach of quantitative and qualitative data collection, organization, and analysis was implored. Quantitative method data collection used case registers to identify mental and neurological disorders among adolescents. Qualitative methods used in-depth interviews of service providers, primary caregivers, and users of healthcare on the services available to treat mental and neurological disorders among adolescents. A combination of quality standards tools was used to assess services.

**Results:**

Epilepsy was the most common treated disorder among adolescents receiving services at the four facilities in the two districts. Providers and stakeholders had limited or no training in adolescent mental health. Validated diagnostic tools were not being used to rule out differential diagnosis; medication procurement was a challenge to consistent treatment. Data collection and analysis was not standardized. Providers, stakeholders, patients, and their primary caregivers reported challenges with funding, transportation logistics, and stigma against people with mental and neurological disorders.

**Conclusion:**

There are few mental health service providers for people living with mental disorders in the two Kintampo districts, with no specific services for adolescents. The Mental Health Act 846 of 2012 is an important milestone in mental health care but there are not specific plans for its implementation. Community sensitization, education in mental health and neurological disorders, and advocacy against stigma are all successful programmes that have the potential to be scaled up.

## Background

World Health Organization (WHO) data estimates that worldwide, more than 450 million people are suffering from mental and neurological disorders. Of these, 350 million people are affected by depression, 60 million by bipolar affective disorder, 21 million people by schizophrenia and psychosis, 47.5 million by dementia, and an unknown number by developmental disorders, such as autism spectrum disorders [1]. WHO also approximates that 50 million people have epilepsy, of which 80% live in low and middle-income countries (LMIC) [2]. Depression, in varying levels of severity, contribute to disability among the general population and severe depression can lead to suicide; globally suicide is a leading cause of death among adolescents [3]. Epilepsy is the most common occurring neurological disorder among adolescents and can continue to impact a person into adulthood [4, 5]. Mental disorders can also coexist with epilepsy and side effects of antiepileptic drugs (AED) can also increase risk for depression [6].

In Ghana, WHO “estimate[s] that of the 21.6 million people living in Ghana, 650,000 are suffering from a severe mental disorder and a further 2,166,000 are suffering from a moderate to mild mental disorder. The treatment gap is 98% of the total population expected to have a mental disorder” [7]. Prevalence of mental illness and its burden among adolescents is unknown on a national level.

In 2012, Ghana passed the Mental Health Act, Act 846, outlining the creation, governance, and management of mental health services at the national, regional and district-levels. The most important outcome was the establishment of the Mental Health Authority. The Mental Health Act however did not include programmatic details of service provision nor any measurable indicators or outcomes for mental health or addressing neurological disorders especially for adolescents at the rural areas. There is no definitive percentage of the health budget set aside for mental health programmes [8]. Though the Act’s provisions ensure alignment with international guidelines of care for children, it does not specify targeted programmes at treating children or adolescents. The use of screening tools or outpatient services aimed at identifying children with CMDs is not mentioned.

In 2007 a move to increase the mental health workforce led to the creation of more community-based mental health workers (CMHW) specializing in mental health [9, 10]. CMHW is composed of Community Psychiatric Officers (CPO), Community Mental Health Officers (CMHO) and Community Psychiatric Nurses (CPN). All roles serve to fill the gap in diagnosis, treatment, and referral of mental disorder cases. In the Brong Ahafo region, several mental health service providers carry out simultaneous programmes to diagnose, treat, and counsel patients with mental disorders (MD). In Ghana, certain neurological disorders like epilepsy are treated by mental health providers.

Few studies have focused on evaluating the current state and impact of mental health service programmes since the implementation of the Mental Health Act after 2012. Of the studies that do, the primary focus is on operations of the entire system [11], workforce retention[9], task-shifting [10] or on adolescents within the greater Accra region only [12]. WHO makes several recommendations on guidelines for child and adolescent mental health policy [13] and guidelines on assessing adolescent health care [14].

There is a growing need to investigate how these service programmes are currently functioning as measured by guidelines from the WHO and tools developed and utilized by past researchers. There is also a need to focus on internal and external challenges faced by these providers in a rural area where access to continuous treatment is especially key in treating mental illness.

## Methods

### Aim of current study

This analysis aimed to investigate the current state of service provision for adolescents with mental and neurological disorders in the Kintampo North and South Districts within the middle belt of Ghana. Specifically, the study objectives were documenting mental and neurological disorders among adolescents seen by mental health service providers in Ghana’s healthcare system; identifying services available for adolescents with mental disorders and neurological disorders; determining perspectives of mental health service providers on the services available for treatment of disorders among adolescents and the quality of those services; ascertaining the perspectives of other stakeholders on treatment and awareness of mental and neurological disorders among adolescents; documenting challenges for service provision for mental and neurological disorders among adolescents; and finally documenting the geo-spatial location of mental health service providers within the study area.

### Study design

This study used a mixed method approach to answer the study objectives. Quantitative methods were used to ascertain the number and descriptive statistics of mental disorders as captured in the case register of the four mental health providers within the two districts, Kintampo North and Kintampo South. Qualitative methods were used to explore the perspectives of conventional medical service providers, primary caregivers, and users of healthcare on the services available to prevent, control, treat and create awareness of common mental disorders (CMD) among adolescents (Participant list in Appendix A).

In 2012, a team in the United Kingdom [15] developed a quality standard assessment tool (Appendix B) based on interviews with parents of adolescents with mental illness to identify important qualities in the service and treatment of their children. The standards were further validated by practitioners, providers, and experts in Child and Adolescent Mental Health Services (CAMHS) to further refine the tool to evaluate quality of service. As a result, 10 quality standards under four overarching domains emerged: (1) practice level (indicated by confidentiality); (2) consultation factors (indicated by knowledge, awareness, communication); 3) health visitors (indicated by attitude, continuity of care); and 4) further services (indicated by access and referral). The WHO and UNAIDS also created a quality assessment tool (Appendix C) for general adolescent health services. However, both tools have not been specifically adapted to evaluate adolescent mental health services in rural Ghana.

**Fig. 1:**
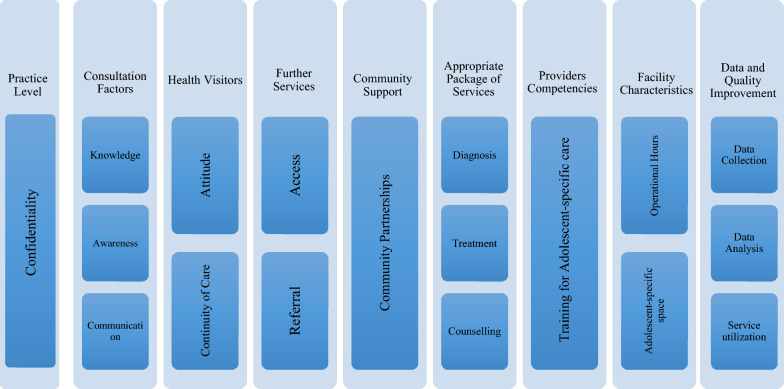
Combined Sayal and WHO Quality Standards and Indicators

In investigating the quality of service provision, the study team adapted the identified quality standard tool by Sayal [15] and the adolescent health standards tool created by WHO and UNAIDS [14] as illustrated in Fig. 1, Appendix D. The quality standard tools as modified by the study team incorporated five of the eight of WHO and UNAIDS’ global quality standards for quality health-care services for adolescents. These five standards by WHO and UNAIDS include community support, appropriate package of services, providers’ competencies, facility characteristics, and data and quality improvement. These standards were adapted into themes and categories to assess health services provided to adolescents with mental and neurological disorders.

A combination of content and thematic analysis was conducted on interviews with all participants using narrative coding and theming data. Primary and secondary categories were based on the quality standards framework. Additional themes that emerged are also included in the final analysis.

### Study setting

**Fig. 2:**
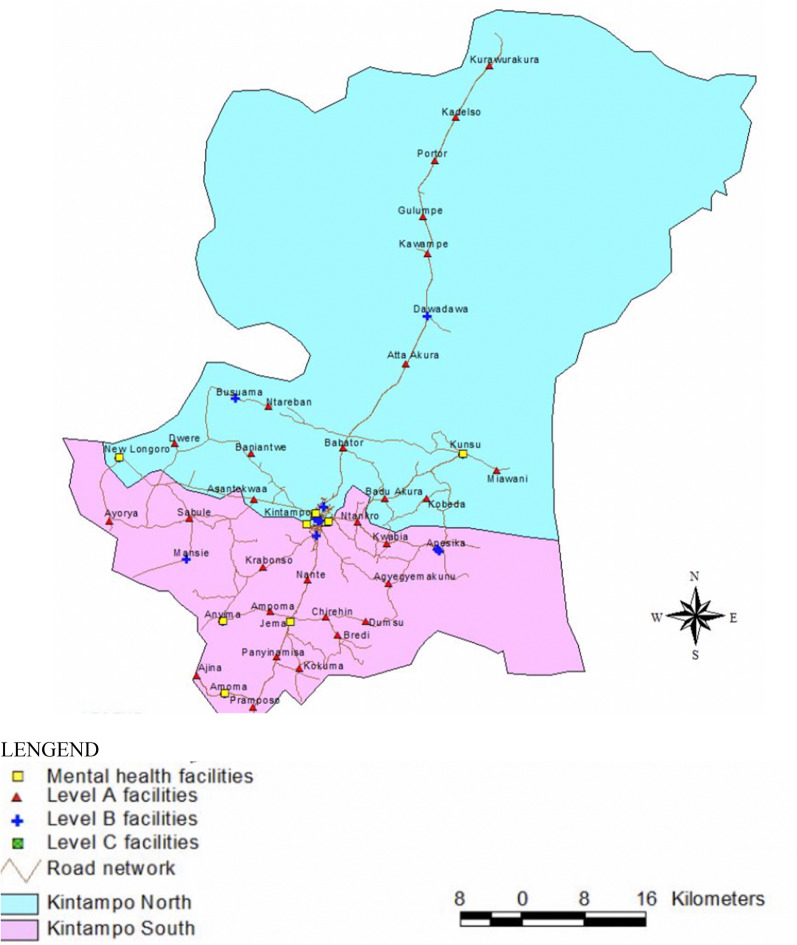
Organization of Mental Health Services in Kintampo North and South

This study was conducted in the Brong Ahafo region, in the two districts of Kintampo North and Kintampo South, both of which lie in the geographical centre of Ghana. There is a total of 12 sub-districts in these two districts. The two Kintampo districts have a total population of about 150,000 and they are predominantly rural (KHDSS, Report 2015) [16]. The majority of the population is the Akan group and its language ‘Twi’ is commonly spoken or understood throughout the two districts. There are two government hospitals, two private hospitals, four health centres, one private clinic, 25 Community Health Planning Services (CHPS) areas and two maternity homes. The College of Health located in Kintampo North is an accredited college that awards diplomas and degrees in clinical health care, public health, and community mental health programmes. A map showing the districts in Ghana is presented in Fig. 2, Appendix D.

### Study population

Mental Health Service Provision in Ghana is a decentralized system and the physical distance between towns and villages requires most providers to spend at least half or some of their time conducting outreach directly in communities. Targeted providers fall under the Community Health Worker (CHW) category. CPOs receive two years of training, can make basic diagnoses and can treat a limited number of mental health conditions. CMHOs receive one year of training and can assist in mental illness detection for referral [9].

Key stakeholders for this study either have a peripheral role in state-run healthcare provision, state mandated social protection, or the public education system. Residents in the study site primarily work in agriculture and farming. According to KHDSS report of 2015, 24.4% of the population, or about 36,000 residents, of the two districts are adolescents. All births are not always registered despite the presence of CHPS compounds. This affects the accurate determination of age, especially of adolescents. Not all children attend formal schooling or continue into higher education after primary school.

### Sampling procedure and data collection

Health and basic demographic characteristics were collected from the case registers of the four service providers in the area. Data was collected over an eight-week period. All transcripts were imported into MAXQDA 12 for data cleaning and coding. The target population for this study included mental health service providers, identified key stakeholders, caregivers of mentally ill adolescents as well as adolescent patients between 10 and 19 years of age.

Health service providers were purposively sampled to partake in a semi-structured interview. This included one Clinical Psychologist, two Community Psychiatric Officers (CPO), one Community Psychiatric Nurse and two Community Mental Health Officers. Other targeted study participants included stakeholders in the implementation of services, such as a Mental Health Authority (MHA) representative, and National Health Insurance Scheme (NHIS) Managers. Also recruited for the study were stakeholders who have professional interactions with adolescents such as School Counsellors, School Health Education Programme (SHEP) Coordinators and the department of Social Welfare.

Participants were contacted directly through mobile phone or visits and gave written consent before participating in the interviews. Interviews were conducted in English and then transcribed. All interviews were digitally recorded in person with two recording devices and lasted 30–60 min.

All participant forms were collected by the study team and kept in the study file in a secure location. All electronic files with identifying information were also kept in a secure, password protected location.

Primary caregivers were recruited purposively from members of an association of caregivers at the Psychosocial Centre. To ensure respondents would give accurate and unbiased responses, the Focus Group Discussion (FGD) took place on the premises of the Kintampo Health Research Centre (KHRC) without the Psychosocial Centre staff. The FGD lasted exactly one hour and was conducted in Twi in early October 2016. After participants signed and gave consent to be interviewed, the discussion which lasted 30–60 min was digitally recorded.

Adolescent users identified for this study (Table 1, Appendix D) have had to at least complete one visit with one of the identified providers and have at least one diagnosis of a mental or neurological disorder either in the past or at present. Adolescent respondents also had to be verbally communicative, in a state lucid and healthy enough to participate (as identified by service providers), and willing to give signed consent along with parental or guardian consent. All parents or guardians were given verbal explanation and interpretation (in Twi) of the written consent form, the study purpose and the reason for interview request. Consent forms were then signed by the parent or guardian, or a thumb print was accepted as a signature on behalf of the child.

### Geo-spatial data

KHRC has Geographic Information System (GIS) data of all Health facilities within the study area (Fig. 2, Appendix D). This information was used for the geo-spatial analysis. GIS coordinates of other subsidiary mental health providers were also collected.

## Results

### Mental Health Service provision in study area

**Fig. 3:**
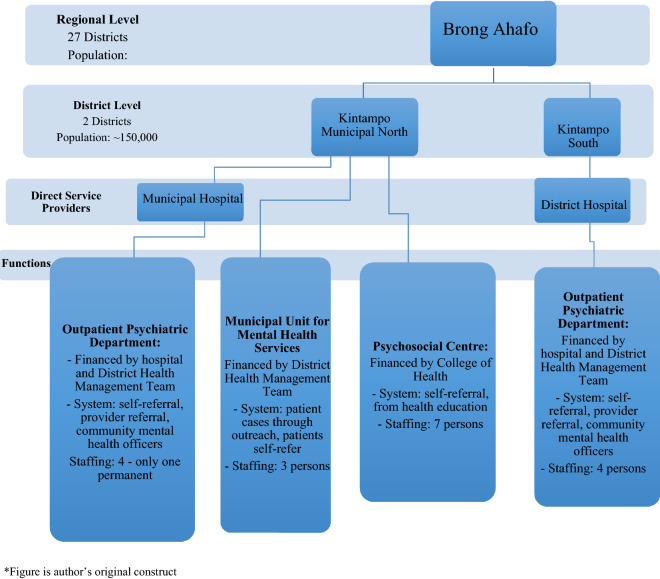
Mental Health Facilities

Mental health services are provided by the two districts’ hospitals as well as the Psychosocial Centre in Kintampo North that serves both districts. Community mental health outreach activities currently take place within 12 sub-districts. The Kintampo Health and Demographic Surveillance System (KHDSS) monitors the population and health dynamics of the population within these two districts [16]. Within the general population, it is estimated that adolescents constitute about 24.4% of the entire population covered by the Kintampo Health and Demographic Surveillance System (KHDSS, Report 2015). The KHDSS has a case register of CMD that is updated periodically with information from the community psychiatric nurse (it includes diagnosis). Figure 3 (Appendix D) shows the organisation of mental health services in the study setting.

### Prevalence of Adolescent Mental Disorders Registered by the service providers

Information of adolescents with mental health problems were recorded differently by all four providers that participated in the study. Some case registers were missing vital demographic features such as date of birth and home town or village, or data was not captured for some of the years. Many of the highest occurring disorders varied in ranking but neurological conditions such as epilepsy and generalized seizure disorder, enuresis (bed-wetting), some form of schizophrenia or psychosis, anxiety disorders and then depressive disorders.

Table 2 (Appendix D) includes the total number of adolescents and combined diagnoses in 2015 within the study area. Epilepsy was the highest reported condition among all adolescent patients, followed by Enuresis and then Anxiety. This was reflected in both female and male patients, where more than 70% were treated for epilepsy and cases of Enuresis occurred only among adolescents less than 15 years old. These facilities are required to report its patient numbers and monthly records to the regional Mental Health Authority representative.

Table 3 (Appendix D) shows is a facility within the outpatient department at the Kintampo Municipal Hospital and currently staffs one full-time CPO and two National Service personnel. Case records are managed by the facility and collected according to age group and gender. This facility’s records combined children, adolescents, and young adults as a single group, from age 5–25. Within the 11 months of patient records, Epilepsy was the highest reported disorder being treated, followed by Schizophrenia, Substance Induced Psychosis, Depression, and Bipolar Disorder. There is a slight variation between female patients and male patients as more females were being treated for depression (5%) than bipolar disorder (1%) than males, 2% and 3% respectively.

Table 4 (Appendix D) is a summation of adolescent mental and neurological disorders as recorded by a facility in Kintampo South. The facility employs three CMHOs, three CPNs, and one National Service personnel. Epilepsy was the most common occurring disorder among adolescents at 50 patients or 69.4%, followed by Anxiety and Psychosis at 11.1%. Between female and male patients however, Anxiety was higher in females at 14% than males at 8%, and Psychosis was a higher occurrence in males at 16% than females at 6%. Both sexes experienced Enuresis as the third most common disorder for 6% of female patients and 11% of male patients.

#### Primary roles, responsibilities, and training

Key summary of the qualitative findings is presented in Appendix E. Nearly all of the healthcare service providers have only been working in their field for a few years, the longest term of any provider being about seven years and the shortest term being three months. When asked about primary roles and responsibilities, all providers discussed tasks mandated by their unit such as outpatient care and educating patients and caregivers on their conditions. Those providers who did receive training did not usually have any specialization in child and adolescent mental health provision beyond basic child psychology.

“We had general training but we were also exposed to different modules so [students] were allowed to go deep into modules that they thought were very helpful [but] we were not exposed to so many of the modules. [The courses included] adolescent mental health and how to handle but that was not as deep as [the students] wanted it, or it should have been.” – Medical provider in the study area.

Stakeholders in the Ghana Education Service and Social Welfare departments tended to have varied experience or training in working with adolescent health issues. Stakeholders in education reported only to have knowledge in addressing reproductive and sexual health, water and sanitation, illegal drug use, or truancy. Training in mental health, neurological health, and emotional wellbeing was not a mandatory component of training received. One stakeholder who works in an administrative educational role admitted to having little or no training and could only identify abnormal behaviour:

“As for adolescents, mental disorders I don’t know what specific disorder I can talk about apart from the fact that behaviours could be seen as odd. We don’t [have] the expertise to judge them, we just see those children as odd ones. Their behaviours are odd but we don’t have any justification to classify them as maybe having mental challenges or having mental disorders.” –Ghana Education Service Representative.

Stakeholders in the National Health Insurance Scheme (NHIS) and the Department for Social Welfare and Community Development, had little to no knowledge in adolescent health, mental health or neurological disorders. These stakeholders often relied on healthcare providers for expertise in determining the health status of a patient or client.

#### Confidentiality

All direct health service providers agreed confidentiality was a core tenant of protecting patient rights and health. Only one provider had visibly posted literature regarding patient confidentiality and the Patient Rights Charter in that facility. All providers verbally communicated to their patients that confidentiality was part of their care. Both health service providers and stakeholders alluded to confidentiality in their respective fields’ Codes of Ethics, but no participant specifically mentioned the Patient Rights Charter, nor other mandated confidentiality policies in their department or agency. Clients’ illiteracy was considered a major barrier to creating and producing literature related to patient privacy, confidentiality or rights.

Both caregivers and patients reported that providers allowed them privacy as most conversations and meeting occurred in a one-on-one environment. It was not reported that any signage or literature was made available to patients or caregivers regarding confidentiality policies.

One patient was asked if it was ensured he was the only one present during consultations with doctor and responded, *“Yes, I am the only person.” – Male, 19, Adolescent Mental Health User.*

#### Consultation, communication, and community sensitization

Participants were asked about components of practice, including how they communicate with clients and caregivers, and if the community was aware of their departments’ services.

##### Consultation and communication

All health service providers stated they often communicated with patients in their dialect or language of choice. The patient’s age often also affected the communication. All of the health service providers found it easier to communicate with teenagers and adolescents when parents or caregivers were not present during the initial meeting. Health providers emphasized the use of education to help patients and their caregivers understand the causes and symptoms of mental disorders.

When asked if the community was aware of services being provided in their units, providers stated they went beyond simply telling patients they encountered but through also education of neighbours and various community groups, including churches.

“We educate and we also treat cases in the community, others see us and they, they take our phone numbers, so if there’s any [need], they just call [us].” – Medical provider in the study area.

Primary caregivers and patients reported positive and open communication with providers, regarding education toward understanding patients’ conditions and feeling a genuine sense of the provider wanting to help the patients.

“What he does is that he also talks to the patients about the wellbeing and how they are responding to the medication that they are on. His nature also allows us to talk to him about anything that is bothering us with regards to our relative’s condition.” – Primary Caregiver, Male.

##### Community sensitization

Community sensitization was a way to create awareness of programmes and services available by providers and by stakeholders. It is also a key tactic for National Health Insurance Scheme (NHIS) representatives and social workers who spent a majority of their time working directly in communities during outreach or for case work. Registration for the NHIS and for the Livelihood Empowerment Against Poverty (LEAP) social welfare programme require support from the community, as both the NHIS and LEAP are mandated to offer protection to persons considered to be mentally ill or living with a form of physical or mental disability.

“That is the sensitization we’ve been doing. We go to [churches in other towns]. Apart from the mental illness they will have other conditions like malaria. Any other condition can come and they need healthcare, so this is what we are basically doing. We also impress upon our Assembly persons, community opinion. If there’s like the chiefs, if they identify somebody in the community as having a mental challenge, the Assembly man will just link up with us.” – NHIS Representative.

The sensitization of community members also prompted many primary caregivers to be more understanding of mental illness and other disorders. At least one primary caregiver reported feeling like an advocate in the community after receiving education from the providers.

“The teachings that they are providing for us is adequate because they have given the training such that even within our neighbourhood if something similar should occur we are able to advise them on what to do.” – Male Primary Caregiver of an Adolescent.

#### Continuity of care, referrals, and partnerships

Providers were also asked about their departments’ protocol on continued care, referral mechanisms, and any existing partnerships with other government or non-governmental agencies. Health service providers reported their facilities’ lack enough personnel and funding to ensure total continuity of care or relied heavily on community mental health workers embedded in the community.

“In fact, I need to go there to supervise [the CMHOs in my department] but I do not have means of transport to go [to those outer villages]. The supervision should have been one of my responsibilities but because of means of transport I cannot go. We need to be going to the [distant villages] to conduct outreach programmes. We have to organize the outreach programmes and all involves money and means of transportation.” – Medical provider in study area.

Some providers were able to connect and work with community partners in handling cases that included socio-economic issues, such as truancy and poverty. One health provider expressed weak ties to non-governmental agencies or other government agencies, whereas the other providers actively sought advice of other health experts.

“[With] referral services, if the condition is beyond my knowledge, then we send them to Sunyani; either Sunyani or National Psychiatric Hospitals. I also have the numbers of various Psychiatric Doctors.” – Medical provider in study area.

Adolescent patients reported they felt their care was being handled as part of an ongoing treatment plan and relationship with their care team. Caregivers also observed consistency in care and constant communication.

“It has not been a long time because from time to time [they] come and visit us here in our household. She recently met one of the health workers on her way to school and they had a conversation.” – Female Primary Caregiver of an Adolescent.

##### Referrals

Stakeholders in Kintampo South tended to work collaboratively with other government agencies. A referral mechanism set up between the health service provider, Department of Social Welfare, and NHIS allows clients to be referred appropriately for their needed services.

“When we identify them, we make or we alert the mental health worker to be aware that we identify this person in this community so if you have any help, attend to the person.” – Social Welfare Officer.

#### Package of services: diagnostics, treatment and counselling

Providers were asked about diagnostic services, medication or other prescribed treatments, and counselling, and if they felt the services available were adequate or appropriate. There were mixed responses from the providers regarding diagnostic services available in their unit, as some pointed out lack of available tests to rule out any biological reasons for patients exhibiting symptoms of mental disorders or epilepsy.

##### Diagnosis

“We cannot [say] it is really appropriate because we lack so many, so many diagnostic tools. We lack so many diagnostic tools so sometimes we look at the signs and symptoms and give a provisional diagnosis and then we treat.” – Medical provider in study area.

“Well, taking a proper history of a client and that history leading you to get your diagnosis, is one of the best way [s] of getting your diagnosis. You know for sure something like epilepsy, which is very common among my clients. It take[s], seeing the person, and the way the person even presents himself. You get that actually it’s epilepsy. Unless probably there are other conditions that are not common.” – Medical provider in study area.

In practice, no provider specified the use of any diagnostic tools or questionnaires for mental illnesses. Two providers did mention the use and necessity of referring to other health professionals for second opinions but this was not a standard practice for providers who worked primarily in the community, such as the CMHOs. Only one provider stated it was standard practice to have patients complete blood tests to rule out alternative causes of specific disorders.

##### Treatment

Providers reported that lack of medications was a difficulty in providing adequate treatment while one cited lack of proper diagnostics serving as a barrier to effective treatment.

“Because we lack effective diagnostic tools so we cannot also not say the treatment is effective but we do our best. We all doing our best to minimize the case prevalence.” – Medical provider, Kintampo North.

Patients perceived effectiveness in medication meant treatment overall was adequate. Primary caregivers reported satisfaction with treatment and access to medications.

“I am happy with the kind of service that they provide here. They have made us understand that their medication reacts slowly; it is only after two weeks that you will feel the reaction of the medication. They also told us to exercise patience when the start reacting towards the medication. I think that he has received all the help that he is supposed to get here.” – Male, Primary Caregiver of an Adolescent.

##### Counselling

Providers viewed counselling as all-inclusive and focused on education, advocacy, and family counselling. One provider mentioned loss to follow up as a challenge in providing counselling services.

“We do counselling. Apart from giving the medication you counsel the person on the condition, and then the primary caregiver, you try to talk to them too. If you explain to [caregivers], they will begin to understand that it’s not intentional, or it’s not because they don’t respect, but it’s a condition that we are trying to manage.” – Medical provider in study area.

Primary caregivers reported counselling services were adequate as they felt included in the process of recovery of relatives. Adolescent patients also expressed counselling services were adequate however two patients did express hesitation in openly discussing their treatment options with their providers.

“The medication that they gave me that I did not see any improvement in my condition was not changed. I was hoping that they will change that medication for me but it did not happen.” When the patient was asked if he has ever reported this to his care team, he replied, “No, I did not say anything to them.” –Adolescent user, Male, 14.

#### Facility characteristics

Providers and stakeholders were asked about operational hours and if their facilities included spaces specifically set aside for adolescents.

##### Operational hours

All providers reported operational hours to be within the average work week of Monday to Friday, from eight or nine in the morning to early evening but in practice, all providers were essentially on-call.

“You cannot get an emergency client and say that you’ve closed, for that matter, you [have to] attend to the client.” – CMHO, Kintampo North

Patients and primary caregivers alike did not seem to know the facility hours but were aware that the facilities were open early in the morning, and that providers were on-call and reachable by phone.

Stakeholders also reported similar official hours of operation but tended to close operations when “the job was done.”

“Normally but you see we go beyond [eight to five] depending on the number of people we have here. If there are people we just work, we make sure we register also, we finish all of them before we leave here. So, there is no limit. Even though it’s eight to five. We go beyond that. If people are here, we don’t leave them like that.” – NHIS Representative, Kintampo South.

##### Adolescent-specific spaces

All of the providers and stakeholders reported not having a specific space within their facilities for children or adolescents. One provider reported a space at their facility had been designated to be for children and adolescents but had been stalled. Another provider in the study area reported she did not foresee being allocated a specific space or area for consultations or treatment of adolescents. A provider working in the facility attached to the Kintampo North reported bed shortages and inpatients with psychiatric issues were admitted in the general ward.

“If the person comes and he or she needs admission you have to mix with the general ward, you have to mix it with the general conditions…It works because there is no option… We calm [aggressive patients]; we give them Anti-psychotics.” – Medical Provider, Kintampo North.

Primary caregivers and patients reported not knowing if a space was designated for children or adolescents but that all appointments occurred in the single facility or took place during home visits.

“I have not noticed a thing like that but every time when we come we meet the doctor [health worker] and we talk to him and he gives us our medication and then we go.” – Male, Primary Caregiver of an Adolescent.

Stakeholders in NHIS and Social Welfare did not have designated spaces for adolescents, preferring instead to either prioritize children and adolescents in registration, or by meeting adolescents and children in their regular offices. Stakeholders in the Ghana Education Service (GES) either used their offices for meetings with students or most often, would refer students to the Adolescent Corners for health issues.

#### Data collection, data analysis, and service utilization

Providers and stakeholders were asked about case registers, reports, data collection, any analysis of the data, and increases or decreases in service utilization by any of their patients and clients.

##### Data collection

Providers reported that they compiled data monthly, which was sent to either the Municipal Health Directorate, Regional headquarters, or kept in-house. Case registers for all four facilities was often compiled into one record of the general population. Each facility had a varied method of data collection and no standardized method of keeping records.

One provider grouped children and adolescents into age cohorts; another provider grouped all children with young adults until the age of 25. Two providers used a computer for data entry. Only one of these providers used an electronic health record system. The other provider used a basic Microsoft Excel spreadsheet for data entry; this file was accessible to all the providers at the facility. This provider acknowledged the difficulty in using a basic system of record keeping.

“We barely had a computer when we started so everything was stored in folders and even the store room where we kept our folders and other information relating to patients is currently inadequate.” – Medical Provider in the study area.

Stakeholders also were tasked with compiling monthly reports. NHIS representatives were given annual and monthly targets for registration of patients according to the district demographics. Social welfare and development officers sent reports for their three designated areas to the district and national headquarters. GES representatives, specifically School Health Education Programme (SHEP) coordinators, were tasked with reporting monthly cases and numbers as part of an ongoing Adolescent Health project funded by an external US organization.

##### Data analysis and service utilization

Providers and stakeholders were asked if any quantitative or qualitative analysis was done on the reports to gauge the successes or failures of programmes. Providers as a whole did not report conducting any in-depth analysis.

Service utilization varied by provider but at least one considered a lack of personnel to be a challenge for utilizing of services.

“There’s more room for improvement but I think the hindrance is lack of personnel. We have drawn up so many programmes, but we need more hands to implement these programme, so I think there’s more room to expand the services for adolescents but in the near future, we will roll out the specific programmes targeting [adolescents], yes.” – Medical provider in the study area.

Stakeholders did not have access to the analyses done on their monthly reports and so did not track service utilization. Stakeholders in NHIS specifically reported analyses were conducted at the national level or were done by other agencies and so did not focus any energies on service utilization.

### Challenges

Providers and stakeholders were finally asked about challenges they faced in carrying out their duties, conducting facility administrative tasks, and following through on mandated protocols. The most common challenges that arose were stigma, transportation logistics, Funding, and medication procurement.

#### Stigma

Providers faced stigma from members of the community and encountered heightened sensitivities from patients who are reluctant to report or seek treatment. Primary caregivers reported experiencing stigma from their neighbours. One primary caregiver reported his own family tended to stigmatize his son as they did not adequately ensure his treatment adherence.

“That one they have been pointing fingers at us that, our relative is mad. Some families may even say the person is a witch or something.” – Female Primary Caregiver of an Adolescent.

Stakeholders reported keeping a high value on client privacy and gaining trust from clients, so a key challenge was finding ways to work around the stigma associated with mental and neurological disorders.

“People don’t want to be tagged that his or her son or daughter is mentally ill, so they probably don’t want to be selected or something like that. That’s the challenge.” – NHIS representative, Kintampo North.

#### Transportation logistics

Every provider and stakeholder reported access to working vehicles such as cars, motorbikes, and motorized scooters, fuel, and regular maintenance as a challenge. Providers reported difficulties in medical and medication compliance when they were unable to physically conduct outreach in the community. All providers reported using their own personal funds for transportation for outreach and administrative tasks.

“I’m using my own motorbike, I buy my own fuel and I just do the field work.” – Medical Provider in the study area

Stakeholders also reported the same issues with transportation and also mentioned that being immobile made it difficult for clients living in remote areas to reach their offices. For practical reasons, some of the stakeholders’ offices need to stay in their offices for network connectivity or in towns where network connectivity was possible.

“You see especially those violent ones. They will not want to put them in those commercial vehicle. Even if it all then charter a taxi and bring only that person, the person may not be fine. We on our part we are trying to eliminate already that we don’t [have] network [s available all] around so we are setting centres closer, registration centres closer to them. And then where possible, we rather bus the people to the registration centre, so that we try [to] sort of eliminate that sort of challenge.” – NHIS Representative, Kintampo South.

#### Funding

Providers and stakeholders reported underfunding as a common challenge that directly impacted areas of practice and administration. Providers reported a lack of priority for funding mental health services as a major road block to providing adequate services overall, including adolescents.

“If you are going to provide the certain special service for adolescents, it all boil[s] down to finances.” –CPN, Kintampo North

One provider reported her unit overall was given low priority and was only given forty Cedis (about ten US dollars) per quarter (about three months) for fuel.

“We don’t generate money. So sometimes you just place your situation before them but we’ve been able to achieve something at least… And then fuel too, it’s inadequate, from my directorate. Imagine Forty Cedis. Which community will I stop going [to]?” – Medical Provider in the study area.

Stakeholders also reported a similar issue as the funding source tended to only come from one place such as the directorate, or regional bodies. A stakeholder in the Social Welfare and Community Development Department was allocated a meagre amount to operate the Department for a quarter of a year.

“We don’t get funding apart from the central government and the central government funding [is] very inadequate. For example, giving a department nine Ghana Cedis. We cannot even buy a paper.” –Social Welfare Officer in the study area.

Representatives from the GES also reported a level of uncertainty after the health club programme funding ends and schools no longer have a way to sustain health education.

“So what we are saying, we want [to maintain] the sustainability of the project [that we feel] is very, very necessary. The project will soon end. Because they give us three years and this is the third year. If they don't extend it, I think the project will end.” – GES Representative, Kintampo South.

Primary caregivers reported financial hardship and personal costs as the largest burden in caring for their relatives. They also reported that despite receiving assistance from NHIS through registration of those suffering from mental illness, this assistance was inadequate and caregivers were still awaiting assistance from other agencies.

“No, they say the [LEAP stipend] is not ready. No there is no place to go, except if someone meets you and they decide to give some money. Also we were informed the boss at the Human Rights [department] is also supposed to make arrangements to get some help for people like us. That one too we do not know when it will come.” – Female Primary Caregiver of an Adolescent.

#### Medication procurement

An issue that arose in interviews with providers was the erratic supply of psychotropic medications. Drugs for patients with mental and neurological disorders are considered Programme Drugs, which means they are to be issued free to patients. However, a shortage in regional medical stores have forced some providers to collect “tokens” (similar to a copay) to obtain drugs. This was a practice reported by all of the providers.

“In fact, we do not generate any funding but what we do is that if we need any drug we go to medical stores in Sunyani. But sometimes when we go we don’t get anything. We also need the clinic to run. Because of that what we did was that we called all the families of the clients, we met here, and then I told them that now when we go to Sunyani we don’t get the drugs so [I asked] what can we do. Then someone suggested that… if they can contribute some money so that we buy some from the drugstore.” – Medical Provider in study area.

There are instances where the inability to pay this token affected the treatment they received at the facility. A provider in Kintampo South recounted a case of a teenage boy with an unspecified disorder whose primary caregiver could not afford to pay the token, and the patient stopped taking his medication. The caregiver eventually decided to pay a token toward the boy’s treatment following an incident.

### Geo-spatial analysis

A map containing the location of all health facilities (including those who provide mental health services) was produced by staff at KHRC using previously available Geospatial data, and estimated distance to the nearest mental health services. The map below illustrates the two districts, Kintampo North and Kintampo South, and all of the available facilities for mental health and neurological disorders.

Level A facilities, 14 of which are located in Kintampo North and 15 of which are located in Kintampo South, are Community-based Health Planning Services (CHPS) compounds. 8 Level B facilities are located in both districts and are health centres that employ midwives and basic health services. Level C facilities are primary care facilities, contain specialty wards, labs, and testing centres, and inpatient units. Level C facilities such as the Kintampo Municipal Hospital and the Kintampo South District Hospital are not visible on the map as they are also considered Mental Health facilities.

## Discussion

Mental health service providers in the study area are tasked with treating several disorders and conditions beyond the capacity of the system, as is a common issue in many lower and middle income countries [17]. Case registers from all providers showed a high occurrence of epilepsy and seizure unspecified with at least 65% among the total number of adolescents. The other 30% or so of diagnoses made at the four facilities included elimination disorders (enuresis), schizophrenic and psychotic disorders, common mental disorders, substance abuse, and migraine. There was a considerably high numbers of providers within the study area (n = 15), as compared to other LMIC in the African region [18], which improved overall outreach and awareness of mental illness.

Providers received minimal education specific to children and adolescent development and psychology. This lack of overall training in child and adolescent mental health for clinical providers and community level providers is an ongoing issue in the African region, along with a shortage of physicians who treat mental illness as an integrated part of primary healthcare [18, 19]. Stakeholders in NHIS, Social Welfare, and GES overall did not report formal training in handling or addressing mental health issues specific to children and adolescents. All of the stakeholders acknowledged the gaps in adolescent specific mental health care and expressed a desire to learn more. This interest in focusing on adolescent mental health, especially as it relates to education, poverty, economic contribution, and advocacy, illustrates a willingness of various actors to collaborate. Stakeholders can importantly serve as valuable ancillaries in health referrals, mental illness interventions, poverty interventions, and in scaling services [20].

Confidentiality and patient privacy were considered important by all participants but no physical spaces for the treatment of adolescents with mental illness were available in the study area to ensure total confidentiality. The Mental Health Act, Sect. 61.2 requires patients be examined in private and not in the view of observers [8]. Most facilities had typical business operational hours and were geographically located within walking distance of several schools in the area. However, spaces for treatment and services for adolescent mental health were limited. Minors and adolescent care was often combined with adult mental health services, including in inpatient settings, counter to WHO and UNAIDS recommendation that adolescents receive appropriate care in appropriate spaces [14]. Stakeholders in NHIS, Social Welfare, and GES reported privacy as a core tenant of service delivery, especially among youth, and they expressed interest in prioritizing this population by ensuring cases were referred to the appropriate agencies. They additionally reported having limited or no dedicated spaces to serve adolescent-specific needs in the context of health referrals.

Communication and community sensitization were priorities for providers and stakeholders alike as they worked to include advocates at the community and to educate primary caregivers. These programmes in community sensitization could be scalable and repeatable by all providers. The use of education campaigns for the public has previously been suggested as a way to reduce stigma [21].

Continuity of care and follow-up by providers with their respective patients was considered appropriate and satisfactory by patients and primary caregivers. A patient-led treatment plan, that is meeting patients’ or caregivers’ expectations, however does not assist in determining overall quality [22]. Despite the overwhelming positive attitude of primary caregivers in the study, there is still a need to utilize meaningful, validated, and quantifiable tools for providers and policy makers to determine quality assurance [22].

Diagnosing conditions appropriately and accurately was another challenge within the study area. Without reliable screening and tests to ensure patients’ proper diagnoses, it is possible some patients were not being given the most effective treatment. Improper diagnostics and care processes can also contribute to overcrowding and lengthy inpatient stays in hospitals, especially those in urban areas of Ghana [23]. Cost and time are often barriers to ensuring patients are given all physiological tests to confirm or refute mental illness [24], an issue in Ghana as well as in other regions of Africa where mental health policies are still in development [25, 26].

Providers lacked specific standards of administrative practices such as standardized record keeping and data entry; invariably, this skewed overall data of the number of cases being treated in each facility. Several data fields were missing, such as the onset of illness, or totally inconsistent, including multiple spellings of one patient’s name (such as reversing given names and surnames) and patients with varying ages within the same year, with differences of up to 2 years.

Underfunding and low prioritisation of mental health services overall affected both providers and stakeholders in addressing adolescents with mental and neurological disorders. This has consistent with previous literature citing a lack of services for those living outside of urban areas. Lack of resources, including staffing, transportation, and administrative tools, in the study area and in similar regions in Africa undermines providers’ ability to manage large caseloads and provide high quality care to all of their clients [22, 27].

Medication procurement arose as a challenge by all providers. Providers reported a shortfall of units of specific medications or unavailability of medications for some of the months, which has been previously reported as a recurring issue [24]. Patients and their caregivers were sometimes given the option to receive a prescription and pay for and acquire medications themselves; cost-prohibitive treatment runs counter to goal of the Mental Health Act, which ensures free mental health care, and further exacerbates health inequities [24]. This challenge is common in several countries and regions, and disruptions in the supply chain of medications and lack of financing compounds the existing challenges patients and caregivers face [21].

The GIS analysis also illustrates that despite the expanse of CHPS compounds, and other basic care clinics, only 8 facilities offer mental health services to a population of about 150,000. Though there is a high number of providers within the total population of the study area, the concentration of services on the border of the two districts means rural communities are given limited access, which further creates a larger burden on providers attempting to provide outreach to very rural towns.

## Study strengths and limitations

The study team did encounter a number of limitations, including poor record keeping, time constraints, and difficulty scheduling interviews with all targeted participants, mainly adolescent users of mental health services. Case registers were requested from all four facilities, but patient records were missing vital data such as date of birth, patient residence, date of onset of illness, and other demographic information. Discrepancies in this data resulted the exclusion of a large number of cases.

Recruitment of primary caregivers and patients was completed through one facility, although these carers does seek healthcare services from the others facilities. There was a high possibility of sampling bias among the participants as members of the Caregivers Association are proactive, and generally supported by the establishment of the group.

Another limitation of this study is the use of the Sayal tool to assessed the quality of adolescent mental health service provision in the area. The Sayal tool has not been validated in the study setting. However, the tool is a guideline document for assessing the current treatment environment, available resources, and how patients and families responded to treatment provided in the area. Although the quality standards tool by Sayal and team was initially developed through several processes that included the involvement of adolescent patients' parents and guardians in the UK, the advantage of the tool is that most questions were adaptable and provided flexibility with language translation and establishing a baseline for healthcare providers and non-health stakeholders. Our study population had varying mental health literacy; nonetheless, we realized that the tool could be administered to participants with limited formal education. Also, some of the probes in the tool had to be re-phrased for participants with no formal education to understand and provide responses. Several of the questions, for example, were subjective and asked for parents’ and guardians’ perceptions of care and providers’ communication style. Often, respondents were unable to choose providers, so no comparisons of better or worse practices could be made. The tool is adolescent, and child-focused who are usually supposed to be treated in a separate unit, but the healthcare providers in this study treated people of all ages because of inadequate resources, especially personnel in the study area. This made it challenging to establish adolescent focused care appropriateness, which we acknowledged as a limitation of the study.

Our study is one of the first to be conducted in this area, and so the findings provide a larger regional view and serve as a baseline for future studies. This tool was adapted and employed for the first time among a population that had not been assessed in any systematic way concerning adolescents' treatment for mental illness. However, as we know that validity is not a property of a tool itself; instead, the interpretation or specific purpose of the assessment tool within a particular setting or population group, our findings are interpreted within the context of the study population and setting. The Sayal tool was the most straightforward and most flexible tool for this study. However, further development or adaptation of this tool would be needed to establish quality standards for populations in low resource areas.

## Conclusion

The results of the situational analysis of mental health services in two rural districts of Ghana show services that are overburdened and overwhelmed by too few personnel attempting to manage several other vulnerabilities of patients with mental disorders with meager resources.

The Mental Health Act of 2012 made important strides in recognizing the need for dignified, holistic treatment of mental illnesses. However, no plans, restrictions, incentives and financing mechanisms have been created to properly implement components of the Act. Adolescent issues and problems, particularly concerning health, are complex and can be often ignored or conflated with other social issues. The involvement of stakeholders such as educators, social welfare thus requires connections not just within the health system, but among policymakers and the local community [28, 29]. Stakeholders from the NHIS, Social Welfare, and Education were not involved in the screening, prevention, or treatment of adolescent mental health in any official capacity. A call for inter-sectoral collaboration as a way to streamline services and efficacy has been proposed in previous studies and reviews [25, 28]. The lack of a standardized protocol or tools for quality measurement can create confusion among providers, what stakeholders can expect from each provider and how caretakers or patients perceive as effective or efficient [22]. Educators involved in school health initiatives received no training in identifying at-risk students or those exhibiting symptoms of mental and neurological disorders, which were often defined as defiance or behavioural issues. Schools are an important arena in which adolescent health and wellbeing can be nurtured and can become a space for early interventions and referrals to health care providers [30].

The practices of educating the community, reducing stigma, and creating a safe reporting environment for mental and neurological disorders should be tracked and analysed for best practices that can be scaled up or reproduced in districts with similar populations.

The safety and health of patients is a top priority but a lack of diagnostic tools, patient charting, and data management means the health workforce may require additional training with newer technologies. This is crucial to efficient and effective treatment and ensuring resources used in providing services is justified.

Further research is needed to find the actual prevalence and incidence of mental disorders and epilepsy in rural areas as these populations are already challenged by low resources and funding and lack of transportation. More quantitative research is needed to find data regarding the adolescent groups in all of Ghana, the role of poverty and social protection in mental and neurological disorders among adolescents, financing of programmes targeted at adolescent health, and improved administrative and data systems.

## Data Availability

The datasets generated and analysed during the current study are not publicly available due the protection of the participants’ anonymity, but are available from the corresponding author on reasonable request.

## Appendix A: List of participants in the study

**Table:**

Service Providers	Personnel
Direct Service providers	1 Clinical Psychologist
	2 CPNs
	2 CMHOs
	1 CPO
Stakeholders	1 MHA Representative, 2 NHI PR officers and NIH Manager
	2 Social Workers
	2 SHEP Coordinators
	1 School Counsellor, 1 Special Education Coordinator
Adolescents	3 adolescents’ participants

## Appendix B: WHO and UNAIDS Global Standards on Health-Care for Adolescents

**Table:**

Adolescents’ health literacy	Standard 1. The health facility implements systems to ensure that adolescents are knowledgeable about their own health, and they know where and when to obtain health services
Community support	Standard 2. The health facility implements systems to ensure that parents, guardians and other community members and community organizations recognize the value of providing health services to adolescents and support such provision and the utilization of services by adolescents
Appropriate package of services	Standard 3. The health facility provides a package of information, counselling, diagnostic, treatment and care services that fulfils the needs of all adolescents. Services are provided in the facility and through referral linkages and outreach.^1^
Providers’ competencies	Standard 4. Health-care providers demonstrate the technical competence required to provide effective health services to adolescents. Both health- care providers and support staff respect, protect and fulfil adolescents’ rights to information, privacy, confidentiality, non-discrimination, non-judgemental attitude and respect
Facility characteristics	Standard 5. The health facility has convenient operating hours, a welcoming and clean environment and maintains privacy and confidentiality. It has the equipment, medicines, supplies and technology needed to ensure effective service provision to adolescents
Equity and non- discrimination	Standard 6. The health facility provides quality services to all adolescents irrespective of their ability to pay, age, sex, marital status, education level, ethnic origin, sexual orientation or other characteristics
Data and quality improvement	Standard 7. The health facility collects, analyses and uses data on service utilization and quality of care, disaggregated by age and sex, to support quality improvement. Health facility staff is supported to participate in continuous quality improvement
Adolescents’ participation	Standard 8. Adolescents are involved in the planning, monitoring and evaluation of health services and in decisions regarding their own care, as well as in certain appropriate aspects of service provision

## Appendix C: Original ten quality standards [15]

**Table:**

	Domain	Quality Standard (QS)
		Quality Indicator (QI)
1	Practice (confidentiality)	QS: GP surgeries should ensure that young people are aware of their policy on confidentiality
		QI: Does the practice advertise its policy on confidentiality (e.g. posters, leaflets, newsletters, practice website)?
2	Consultation (knowledge)	QS: GPs’ knowledge about child and adolescent emotional and behavioural health should remain up to date
		QI: One doctor at the practice should be the lead for work with children and young people
3	Consultation (awareness)	QS: GPs should be aware that parents may need help and advice in managing and coping with their child’s emotional or behavioural difficulties
		QI: GPs should direct parents to information for further advice and to support services
4	Consultation (communication)	QS: GPs should communicate effectively with both parents and children about the child’s emotional and behavioural health
		QI: Survey of parents and children—do they feel the GP communicates well with them about child and adolescent mental health issues?
5	Consultation (communication)	QS: GPs should give parents time to talk during the consultation
		QI: Survey of parents and children—do you feel that the GP gave you time to talk during the consultation?
6	Consultation (communication)	QS: GPs should be able to communicate with children and young people effectively and build good relationships with them
		QI: Survey of children and young people—to obtain their opinion about their experience of speaking to the GP and their relationship with the GP
7	Health Visitors (continuity of care)	QS: Parents should have the choice to see the same Health Visitor in order to establish a relationship where concerns about a child’s emotional or behavioural health can be discussed
		QI: Survey of parents – have you been able to see the same Health Visitor each time?
8	Health Visitors (attitude)	QS: Health Visitors should be non-judgemental when listening to parents’ concerns about their child’s emotional or behavioural health
		QI: Survey of parents – did you feel your Health Visitor was non-judgemental when you spoke about your concerns?
9	Further services (access and referral)	QS: If appropriate, and if they are unable to provide the help required, GPs should respond to parental requests for help for their child’s emotional or behavioural difficulties by making a prompt referral for further advice
		QI: Audit the GP practice – Are referrals made to CAMHS and related services? What is the time interval between the appointment and making the referral?
10	Further services (referral)	QS: GPs should explain the process of a referral and waiting period so that parents feel adequately informed
		QI: Survey of parents of referred children– did you feel the referral process was adequately explained to you?

## Appendix D

See Figs. 1, 2, 3

See Tables 1, 2, 3 and 4

**Table 1:** Demographic Characteristics of the study participants

Caregiver focus group demographics			
Variable		Frequency (%)	
Sex			
Male		2 (18%)	
Female		9 (82%)	
Age			
20–29		0 (0%)	
30–39		1 (9%)	
40–49		2 (18%)	
50–59		4 (36%)	
60–69		3 (27%)	
70–79		1 (9%)	
Occupation			
Farmer		4 (36%)	
Trader		5 (45%)	
Retired		1 (9%)	
Unemployed		1 (9%)	
Relationship to Patient			
Parent		5 (45%)	
Spouse		2 (18%)	
Child		2 (18%)	
Sibling		1 (9%)	
Self		1 (9%)	

Adolescent participant characteristics			
Variable	Frequency	NHIS Registered	Receiving treatment
Sex			
Male	2	Yes	Yes
Female	1	Yes	Yes
Age			
14	1	Yes	Yes
18	1	Yes	Yes
19	1	Yes	Yes
Primary diagnosis			
Schizoaffective disorder	1	Yes	Yes
Epilepsy	1	Yes	Yes
Enuresis	1	Yes	Yes

*NHIS* National Health Insurance Scheme

**Table 2:** Distribution of commonly treated mental disorders among adolescents in 2015 in Kintampo North and South Districts (N = 285)

Combined diagnoses	Total	Female n (%)	Male n (%)
Epilepsy	193	87 (68)	106 (68)
Enuresis	39	17 (13)	22 (14)
Anxiety	19	11 (9)	8 (5)
Migraine	7	3 (2)	4 (3)
Acute psychosis	3	0 (0)	3 (2)
Psychosis	8	2 (2)	6 (4)
Seizure unspecified	7	5 (4)	2 (1)
Enuresis nocturnal	2	0 (0)	2 (1)
Schizoaffective disorder	3	1 (1)	2 (1)
Substance abuse	2	1 (1)	1 (1)
Bipolar disorder	1	1 (1)	0 (0)
ADHD	1	0 (0)	1 (1)
Total	**285**	**128 (100%)**	**157 (100)**

**Table 3:** Distribution of Commonly Treated Disorders Among 5–25-year old between July 2015 and July 2016 In the Kintampo Municipality

Combined diagnoses	Total	Female n (%)	Male n (%)
Epilepsy	250	109 (75)	141 (78)
Schizophrenia	29	13 (9)	16 (9)
Substance induced psychosis	16	6 (4)	10 (6)
Depression	10	7 (5)	3 (2)
Bipolar disorder	7	2 (1)	5 (3)
Other	14	9 (6.1)	5 (2.8)
Total	**326**	**146 (100)**	**180 (100)**

**Table 4:** Distribution of commonly treated disorders among adolescents in 2015 in Kintampo South District (N = 72)

Combined diagnoses	Total	Female n (%)	Male n (%)
Epilepsy	50	26 (74)	24 (65)
Anxiety	8	5 (14)	6 (16)
Psychosis	8	2 (6)	4 (11)
Enuresis	6	2 (6)	3 (8)
Total	72	35 (100)	37 (100)

## Appendix E: Key summary of qualitative findings from service providers, Kintampo

**Table:**

Theme	Key findings
Roles, responsibilities, and training for health providers	Nearly all of the healthcare service providers have only been working in their field for a few years, the longest term of any provider being about seven years and the shortest term being three months. When asked about primary roles and responsibilities, all providers discussed tasks mandated by their unit such as outpatient care and educating patients and caregivers on their conditions. Those providers who did receive training did not usually have any specialisation in child and adolescent mental health provision beyond basic child psychology
Confidentiality	All direct health service providers agreed confidentiality was a core tenant of protecting patient rights and health. Only one provider had visibly posted literature regarding patient confidentiality and the Patient Rights Charter in that facility
Consultation, Communication, and Community Sensitization	All health service providers stated they often communicated with patients in their dialect or language of choice. The patient’s age often also affected the communication. All of the health service providers found it easier to communicate with teenagers and adolescents when parents or caregivers were not present during the initial meeting. Health providers emphasized the use of education to help patients and their caregivers understand the causes and symptoms of mental disorders. Community members not very aware of the adolescent mental health service availability in the health facilities
Continuity of Care, Referrals, and Partnerships	Health service providers reported their facilities’ lack enough personnel and funding to ensure total continuity of care or relied heavily on community mental health workers embedded in the community
Package of Services: Diagnostics, Treatment and Counselling	Providers were asked about diagnostic services, medication or other prescribed treatments, and counselling, and if they felt the services available were adequate or appropriate. There were mixed responses from the providers regarding diagnostic services available in their unit, as some pointed out lack of available tests to rule out any biological reasons for patients exhibiting symptoms of mental disorders or epilepsy
Facility Characteristics and working hours	All providers reported operational hours to be within the average work week of Monday to Friday, from eight or nine in the morning to early evening but in practice, all providers were essentially on-call. There were no spaces designated for adolescents but that all appointments occurred in the single facility or took place during home visits
Data Collection, Data Analysis, and Service Utilisation	Mental health service providers reported that they compiled data monthly, which was sent to either the Municipal Health Directorate, Regional Headquarters, or kept in-house. Each mental health facility had a varied method of data collection and storage, and no standardised method of keeping mental health patients records. Basic data collected from patients are not used for any planning purposes
Challenges for adolescent mental health care in the area	Participants reported that the main challenges inhibiting the provision of adolescents mental health services include; stigma and discrimination, transportation logistics, lack of funding especially for medication procurement for patients
Geo-spatial Analysis	Mapping of the all the mental health facilities in the area with the different level of capacity to operate

## Abbreviations

AED: Anti-epileptic drug
CAMHS: Child and Adolescent Mental Health Services
CHPS: Community Health Planning Services
CHW: Community Health Worker
CMHW: Community Mental Health Worker
CMHO: Community Mental Health Officer
CPO: Community Psychiatric Officer
CPN: Community Psychiatric Nurse
GES: Ghana Education Services
GIS: Geographic Information System
KHDSS: Kintampo Health Demographic Surveillance System
KHRC: Kintampo Health Research Centre
LMIC: Low and Middle-income Countries
MD: Mental Disorders
MHA: Mental Health Authority
NHIS: National Health Insurance Scheme
SHEP: School Health Education Program
WHO: World Health Organization
